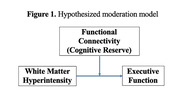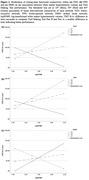# Cognitive reserve: Resting‐state functional connectivity moderates the association between white matter hyperintensity and executive function in older adults with subcortical ischemic vascular cognitive impairment

**DOI:** 10.1002/alz.092227

**Published:** 2025-01-09

**Authors:** Yi Gu, Chun Liang Hsu, Nárlon Cássio Boa Sorte Silva, Roger Tam, Walid Ahmed Alkeridy, Kevin Lam, Teresa Liu‐Ambrose

**Affiliations:** ^1^ Centre for Aging SMART, Vancouver Coastal Health Research Institute, Vancouver, BC Canada; ^2^ University of British Columbia, Vancouver, BC Canada; ^3^ Djavad Mowafaghian Centre for Brain Health, Vancouver, BC Canada; ^4^ The Hong Kong Polytechnic University, Hong Kong Hong Kong; ^5^ King Saud University, Riaydh Saudi Arabia

## Abstract

**Background:**

Subcortical ischemic vascular cognitive impairment (SIVCI) is the most common form of vascular cognitive impairment. White matter hyperintensities (WMH) are hallmarks of SIVCI and are associated with impaired executive function. Cognitive reserve is a property of the brain that moderates an individual’s ability to maintain cognitive performance given brain injury or pathology. The neural correlates of cognitive reserve remain elusive; however, resting‐state functional connectivity (rs‐FC) in networks such as the fronto‐executive network (FEN), fronto‐parietal network (FPN) and default mode network (DMN) have been proposed as potential candidates. The role of these networks in mitigating the impact of SIVCI‐related pathology on cognition remains unclear. Therefore, we investigated whether intra‐network rs‐FC in the FEN, FPN, and DMN moderated the negative impact of WMH on executive function in older adults living with SIVCI.

**Method:**

We conducted a cross‐sectional study among 38 community‐dwelling older adults with SIVCI. Executive function was assessed by the Trail Making Test (B‐A). WMH volume was quantified by T2‐weighted and proton density‐weighted structural magnetic resonance imaging (MRI) using a seed‐based method and log‐transformed before analyses. The rs‐FC was computed via a 5‐min resting‐state functional MRI scan with priori‐selected region‐of‐interest masks. A moderation analysis was conducted to assess whether intra‐network rs‐FC in the FEN, FPN and DMN moderated the association between WMH volume and executive function, adjusting for age, sex, and Montreal Cognitive Assessment (MoCA) score.

**Result:**

The participants had a mean age of 74.1 years (SD = 5.5) and 71% were female; the mean MoCA score was 21.29 (SD = 2.66). Compared with individuals with lower intra‐network rs‐FC of DMN, those with greater intra‐network rs‐FC of DMN (non‐standardized *b* = ‐2913.64, *p* = 0.000) showed better Trail Making Test performance under greater WMH load. In contrast, individuals with lower intra‐network connectivity of FEN (*b* = 2050.34, *p* = 0.007) and FPN (*b* = 1790.81, *p* = 0.005) had better Trail Making Test performance under greater WMH load.

**Conclusion:**

The strength of intra‐network connectivity in the FEN, FPN, and DMN moderates the impact of WMH on executive function in older adults with SIVCI. The results shed light on the potential neural basis of cognitive reserve against WMH.